# The development of a multidimensional Consumer Perceived Value scale in ophthalmology services

**Published:** 2019

**Authors:** Consuela-Mădălina Gheorghe, Victor Lorin Purcărea, Iuliana Raluca Gheorghe

**Affiliations:** * “Carol Davila” University of Medicine and Pharmacy, Department of Marketing and Medical Technology, Bucharest, Romania

**Keywords:** Consumer Perceived Value, ophthalmology services, competitive advantage

## Abstract

**Objective:** The aim of this study was to investigate the dimensionality of Consumer Perceived value in Romanian ophthalmology services.

**Material and Methods:** The sample consisted of 173 consumers of private ophthalmology services, recruited using a systematic method. The design of the study was cross-sectional and the research instrument was a self-administered questionnaire, namely a Consumer Perceived Value Scale, developed by Chahal and Kumari. The dimensions of the Consumer Perceived Value were assessed by conducting an Exploratory Factor Analysis and the scale’s reliability was checked with the Cronbach’s alpha coefficient.

**Results:** The findings of the Exploratory Factor Analysis revealed that all initial factors loaded properly and the Cronbach’s alpha coefficients had values greater than the recommended threshold of 0.70. As such, the Consumer Perceived Value scale had a Cronbach’s alpha coefficient of 0.77 and encompassed the following dimensions: transaction value, aesthetic value, efficiency value, self-gratification value, acquisition value and social interaction value.

**Conclusions:** Applying efficient value strategies in ophthalmology services may ensure consumer satisfaction, loyalty, positive word-of-mouth and offer competitive advantages.

## Introduction

Nowadays, health care organizations are searching for a competitive advantage, as the health care service is not easily grasped by consumers, and at the same time, it is unwanted in comparison with other services [**1**]. Moreover, since consumers became value oriented, health care managers had to look for new methods to support them and grow their interest in the services provided so as a Marketing 3.0 era emerged [**2**]. The Marketing 3.0 era was linked to collaboration, globalization, and creativity, with the outcome of applying successful marketing practices [**3**]. After quality, consumer satisfaction and consumer loyalty, the consumer perceived value (CPV) has received the greatest attention in the strategic marketing. 

The concept of value has been described as having an epistemic role in marketing, and may explain consumer behavior both before and after purchase [**4**]. In other words, value stands at the heart of every exchange relationship [**5**]. Still, Zeithaml defined perceived value as the consumer’s overall assessment of the utility received in comparison to what is given [**6**], and according to Keller, consumers combine the perception of quality with the perception of cost in order to reach a balance in the shape of perceived value [**7**]. Despite the fact that literature on value is as broad and as extensive as possible [**8**], the most encountered characteristic of perceived value is that it represents a trade-off between benefits and sacrifices of consumers when they make a purchase decision [**9**]. Moreover, even if value is well documented and researched by scholars in marketing literature, most of the studies were conducted in developed countries or were confined to the goods sector. In addition, consumers have different perceptions about value because of their differences in preferences and financial abilities. As such, the conceptualization of CPV remains unclear and extremely divergent in literature [**10**]. There are two main approaches related to the conceptualization of consumer perceived value in scientific literature: the first approach describes perceived value, as being related to benefits, be they economic, social, and relational, and the second approach is composed of the sacrifices, be they in terms of price, time, effort, risks, and opportunities [**11**].

Although the CPV concept may be applied in all services sectors, the literature which deals with health care CPV is quite scarce. Accordingly, CPV in health care services is the difference between benefits and sacrifices. In health care services, the benefits are generally the outcome of excellent quality, referring to the process, functional and technical quality, whereas, sacrifices may be embodied by monetary costs and non-monetary costs, such as time spent, mental and physical stress [**12**]. Further, Prahalad and Hamel strongly believe that in health care services, perceived value is in fact a co-creation value, suggesting an “obsessive focus on personalized interactions between the consumer and the organization”, and, specifically, “communities of informed, networked, empowered and active consumers” may trigger challenging changes in value creation as perceived after a medical treatment program [**13**]. Further, Choi et al. [**14**] and Ekrem and Fazil [**15**] concluded that little attention has been given to the CPV scale development in health care services. Thus, in order to have an in-depth insight of the antecedents and consequences of consumer perceived value in health care services, its dimensions should be explored so as to build and maintain long-term relationships between health care organizations and consumers, as well as, determine high levels of consumer satisfaction, trust and positive behavioral intentions.

The aim of this study was to investigate the dimensionality of consumer perceived value in Romanian ophthalmology services. 

## Background

In the systematic review on CPV literature, Sanchez-Fernandez and Iniesta-Bonillo [**16**] identified two research directions related to the dimensionality of the concept. The first research direction, which is widely embraced by the marketing literature, presents the consumer value concept as a unidimensional construct [**6**]. Obviously, the unidimensional approach has proved to be too narrow or simplistic to what consumers might experience in terms of value, while the second research direction is based on a multidimensional perspective [**9**,**17**]. Up to this moment, despite the fact that the multidimensional perceived value construct is not that much explored in the literature [**16**], it is preferred over the unidimensional construct [**4**]. Moreover, the multidimensional approach focuses on an integrative dimensions’ framework, as illustrated in **Table 1**.

**Table 1:** The multidimensional approaches of the perceived value

Year	Dimensions	Reference
1991	- Social value	Sheth et al. [**18**]
	- Emotional value	
	- Functional value	
	- Epistemic value	
	- Conditional value	
	- Cognitive value	Ekrem, Fazil [**15**]
	- Affective value	
1997	- Cognitive value	Grönroos [**19**]
	- Emotional (psychological value)	
1999	- Social value	Sweeney, Soutar, Johnson [**20**]
	- Emotional value	
	- Functional value (price/ value for money)	
	- Functional value (performance/ quality)	
	- Functional value (versatility)	
2001	- Functional dimension (economic and quality)	Sweeney and Soutar [**17**]
	- Social dimension	
	- Emotional dimension	
2006	- Functional value of the establishment (installations)	Sanchez et al [**21**]
	- Functional value of the contact personnel (professionalism)	
	- Functional value of the service purchased (quality)	
	- Functional value (price)	
	- Emotional value	
	- Social value	
*Source: Sanchez et al., p. 396 [**21**]*		

**Fig. 1:**
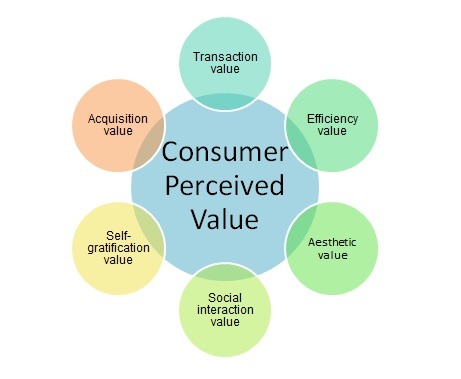
Conceptual framework of the Consumer Perceived Value in Ophthalmology services

Based on a thorough literature review of CPV in health care services, the multidimensional approach comprising the following six constructs [**12**] (**Fig. 1**) was used in this study:

- transaction value consists of the psychological satisfaction gain from the service encounter, as for instance timely services delivered, personalized care, post-medical treatment, good medical advice, prompt response to consumers’ requests;

- efficiency value comprises how effectively and efficiently the service provider delivers the health care services with the help of well-experienced staff, adequate visiting hours, explaining reason for medical problems, treatment;

- aesthetic value is the visual appeal of the health care organization and the ambient condition such as well-dressed employees, clean corridors and washrooms, clean clothing and bedding, proper ventilation;

- social interaction value is shaped by the impartial treatment such as nursing interaction, comfort zone with physician interaction, interaction with society;

- self-gratification value refers to the well-being of patients;

- acquisition value is defined by the overall net value concept.

Accordingly, the hypothesis of the conceptual framework is that the Consumer Perceived Value in ophthalmology services is a multidimensional construct, made up of six dimensions. 

## Material and Methods

**• Participants**

Participants were the ophthalmology service consumers of a private health care organization, located in Bucharest, Romania. The inclusion criteria encompassed individuals who had an ophthalmology routine consultation, wore glasses for more than 3 years, had over 18 years and had no self-reported psychiatric morbidity, or other impairments which would cause difficulty in understanding certain concepts and words. 

The selection of the participants was conducted on a systematic sampling method, suggesting that every third person scheduled for a routine ophthalmologic consultation was asked to take part in the study. Out of 200 initial respondents, 173 agreed to participate. Before filling in the questionnaires, all individuals completed written informed consent forms. As such, out of 173 respondents, the vast majority had the following characteristics: males (53.2%), who graduated primary school (27.2%) and were not married (32.4%). The mean age of the sample was 42.09±7.12. 

**• Procedure**

The study design was cross-sectional and the data collection was performed between May 2019 and August 2019. The research instrument was a self-administered questionnaire which was printed and enclosed in sealable envelopes, so as to ensure confidentiality. 

The questionnaire consisted of two sections:

- First section gathered socio-demographic information of the respondents such as age, gender, education level and marital status;

- The second section comprised the CPV scale, as developed by Chahal and Kumari [**12**] and referred to the 6 dimensions of CPV, namely, efficiency value, aesthetic value, self-gratification value, social interaction value, transaction value and acquisition value. 

All items were measured with 5-point Likert scales, ranging from 1-*Strongly Disagree* to 5-*Strongly Agree*. 

**• Data collection**

The data collection was assessed following a two stage approach for the CPV measurement scale. The first stage consisted of a pilot study conducted on 20 consumers after an ophthalmology routine consultation. Consequently, the items in the questionnaire were checked in order to provide appropriate responses, uncover ambiguous wording or errors. The outcome of the first stage revealed there were no items to be removed from the CPV scale. 

The second stage of the research consisted of the self-administration of the 29 item questionnaire, as depicted in **Table 2**. 

**Table 2:** The CPV measurement scale

Dimension	No. of items	Observations
Acquisition value	4	-
Transaction value	7	2 items are reversed
Efficiency value	5	-
Aesthetic value	6	1 item is reversed
Social interaction value	3	-
Self-gratification value	4	-
*Source: Chahal and Kumari [**12**]*		

• Statistical Analyses

The data was analyzed with SPSS version 20. More exactly, to determine the underlying dimensions of the perceived value construct, an exploratory factor analysis (EFA) with Varimax rotation was conducted. A factor loading of more than 0.40 was treated as being appropriate to be included in the latent factor category. The adequacy of the factor analysis was established by the Kaiser-Meyer-Olkin measure of sampling adequacy and Bartlett’s test of sphericity (p<0.001). 

The reliability test, measured with Cronbach’s alpha coefficient, was used to determine how strong the items of the questionnaire relate to each other. The internal consistency of the scale was deemed to be acceptable if it exceeded the threshold of 0.70 [**22**]. 

The threshold for statistical significance in all tests was p < 0.05.

## Results

The dimensionality of the Consumer Perceived Value in ophthalmology services was determined by the Exploratory Factor Analysis and the Cronbach’s alpha coefficients of the 6 dimensions of the scale. 

The findings of the Exploratory Factor Analysis are illustrated in **Table 3**. All items loaded properly on their initial factors and had values exceeded 0.7. So, the underlying dimensions of the CPV scale in the context of ophthalmology services are made up of the 6 dimensions. The Cronbach’s alpha coefficients of all factors had values greater than 0.7.

**Table 3:** The Exploratory Factor Analysis Results

Factors	Factor Loading	Eigenvalue	Explained Variance	Cronbach’s alpha coefficient
Transaction value		4.7	16.22	0.91
Transaction value_it1_rev	0.838			
Transaction value _it2	0.825			
Transaction value _it3	0.788			
Transaction value _it4	0.797			
Transaction value _it5	0.786			
Transaction value _it6_rev	0.802			
Transaction value _it7	0.851			
Aesthetic value		4.2	14.49	0.91
Aesthetic value_it1	0.838			
Aesthetic value _it2	0.813			
Aesthetic value _it3	0.829			
Aesthetic value _it4	0.820			
Aesthetic value _it5	0.817			
Aesthetic value _it6_rev	0.859			
Efficiency value		3.53	12.20	0.89
Efficiency value_it1	0.827			
Efficiency value _it2	0.831			
Efficiency value _it3	0.846			
Efficiency value _it4	0.843			
Efficiency value _it5	0.814			
Self-gratification value		3.04	10.50	0.88
Self-gratification value_it1	0.886			
Self-gratification value _it2	0.841			
Self-gratification value _it3	0.847			
Self-gratification value _it4	0.860			
Acquisition value		2.81	9.71	0.85
Acquisition value_it1	0.826			
Acquisition value _it2	0.841			
Acquisition value _it3	0.835			
Acquisition value _it4	0.823			
Social interaction value		2.28	7.89	0.83
Social interaction value_it1	0.869			
Social interaction value _it2	0.886			
Social interaction value _it3	0.863			
*Bartlett’s test of Sphericity (p<0.001), Kaiser-Meyer-Olkin=0.816, Cronbach’s alpha=0.77*				

## Discussion

The development of a multidimensional CPV scale in the context of ophthalmology services may have significant theoretical and practical implications. As such, this study extends the literature on consumer perceived value by offering an in-depth insight of its meaning and measurement. Specifically, our study tested a six-item dimensional CPV scale in ophthalmology services. 

Findings revealed that the consumers’ perceived value of ophthalmology services is based on the following dimensions: transaction value, efficiency value, aesthetic value, self-gratification value, acquisition value and social interaction value. In other words, ophthalmology services managers should focus on providing value to their consumers by elaborating efficient strategies based on the six value dimensions. 

Applying efficient value strategies may ensure consumer satisfaction, loyalty, positive word-of-mouth, as well as, offer competitive advantages. Moreover, from a consumer’s point of view, obtaining value is a fundamental purchase goal and it becomes a core element in the exchange process, which may be shaped by several benefits, such as cognitive benefits, reflected in knowledge acquisition, economic benefits, designating monetary compensation, hedonic benefits in enjoyment and excitement, personal benefits suggested by recognition, status and social esteem, practical benefits concerned with real life implications and, last, the social benefits, which are reflected in the established relationships with other consumers. From the organization’s perspective, the most critical source of competitive advantage is the creation of consumer value, which would replace the quality management paradigm [**9**] and it would be the ground for all marketing activities [**23**]. 

**Fig. 2:**
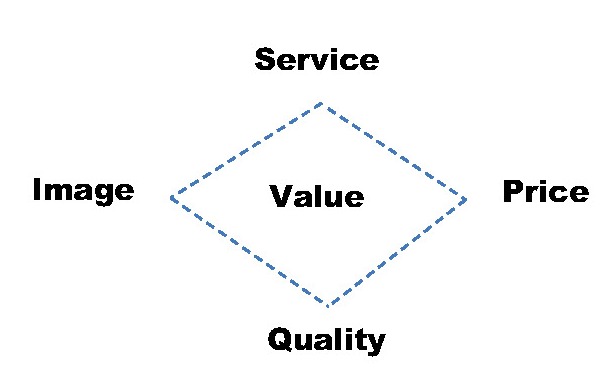
The S-Q-I-P diamond framework. Source: Weistein, p. 51 [**2**] (All rights reserved)

According to Weinstein, the S-Q-I-P diamond framework depicts the antecedents of value, namely, service, image, price and quality [**2**] (**Fig. 2**). The backbone components of an organization’s offerings (quality and service) are represented on the vertical axis of the diamond model, whereas on the horizontal axis, the image and price provide cues for the target audiences. Creating value for consumers through the diamond model will provide a solid business philosophy for the organization, guide all strategic decisions, and trigger positive business performance. 

Value oriented marketing strategies may bring the following advantages to an ophthalmology organization [**2**]:

- Understand consumers’ choices and needs;

- Identify potential consumer segments;

- Increase the competitive options and diminish threats;

- Improve service quality;

- Concentrate on what is meaningful to consumers;

- Build consumer loyalty;

- Develop strong relationships with consumers. 

The limitations of this study emphasize some future research directions. Thus, the focus on private health care services, with specific interest in ophthalmology services, leads to an extension suggestion, namely, to investigate CPV in public health care services as well. Moreover, we encourage the comparison between the private and public health care services in terms of CPV. The generalizability of our findings should be done with caution, as the study sample comprised consumers from Bucharest and we strongly recommend the CPV scale to be validated in other contexts and health care specialties, in order to determine which dimension of perceived value, becomes the most important. In addition, several causal relationships should be empirically assessed, meaning to explore the established relationships between CPV and several other variables, such as service quality, internal marketing strategies, consumer loyalty, consumer satisfaction, external marketing strategies, word-of-mouth, organizational behavior in the context of ophthalmology services.

## Conclusions

The development of a multidimensional CPV scale in the context of ophthalmology services proved to have practical implications in the shape of meaning and measurement. The CPV scale in ophthalmology services was made up of the following dimensions: transaction value, efficiency value, aesthetic value, self-gratification value, acquisition value and social interaction value. 

Elaborating and implementing efficient value strategies in ophthalmology services may increase consumer satisfaction, loyalty, positive word-of-mouth and offer competitive advantages. 
